# Squalomix: shark and ray genome analysis consortium and its data sharing platform

**DOI:** 10.12688/f1000research.123591.1

**Published:** 2022-09-21

**Authors:** Osamu Nishimura, John Rozewicki, Kazuaki Yamaguchi, Kaori Tatsumi, Yuta Ohishi, Tazro Ohta, Masaru Yagura, Taiki Niwa, Chiharu Tanegashima, Akinori Teramura, Shotaro Hirase, Akane Kawaguchi, Milton Tan, Salvatore D'Aniello, Filipe Castro, André Machado, Mitsumasa Koyanagi, Akihisa Terakita, Ryo Misawa, Masayuki Horie, Junna Kawasaki, Takashi Asahida, Atsuko Yamaguchi, Kiyomi Murakumo, Rui Matsumoto, Iker Irisarri, Norio Miyamoto, Atsushi Toyoda, Sho Tanaka, Tatsuya Sakamoto, Yasuko Semba, Shinya Yamauchi, Kazuyuki Yamada, Kiyonori Nishida, Itsuki Kiyatake, Keiichi Sato, Susumu Hyodo, Mitsutaka Kadota, Yoshinobu Uno, Shigehiro Kuraku

**Affiliations:** 1 Laboratory for Phyloinformatics, RIKEN Center for Biosystems Dynamics Research, Kobe, Hyogo, 657-0024, Japan; 2 Joint Support-Center for Data Science Research, Database Center for Life Science, Mishima, Shizuoka, 411-8540, Japan; 3 Molecular Life History Laboratory, National Institute of Genetics, Mishima, Shizuoka, 411-8540, Japan; 4 Department of Genetics, Sokendai (Graduate University for Advanced Studies), Mishima, Shizuoka, Japan; 5 Fisheries Laboratory, Graduate School of Agricultural and Life Sciences, University of Tokyo, Hamamatsu, Shizuoka, 431-0214, Japan; 6 Illinois Natural History Survey, Prairie Research Institute, University of Illinois at Urbana-Champaign, Champaign, Illinois, USA; 7 Biology and Evolution of Marine Organisms, Stazione Zoologica Anton Dohrn, Villa Comunale, Napoli, Italy; 8 Interdisciplinary Centre of Marine and Environmental Research, University of Porto, Porto, Portugal; 9 Faculty of Sciences, University of Porto, Porto, Portugal; 10 Department of Biology, Graduate School of Science, Osaka Metropolitan University, Osaka, Osaka, Japan; 11 Japan Fisheries Research and Education Agency, Hachinohe, Aomori, Japan; 12 Graduate School of Veterinary Science, Osaka Metropolitan University, Izumisano, Osaka, Japan; 13 Waseda Research Institute for Science and Engineering, Waseda University, Tokyo, Japan; 14 School of Marine Biosciences, Kitasato University, Sagamihara, Kanagawa, Japan; 15 Graduate School of Fisheries and Environmental Sciences, Nagasaki University, Nagasaki, Nagasaki, Japan; 16 Okinawa Churaumi Aquarium, Motobu, Okinawa, Japan; 17 Centre for Molecular Biodiversity Research, Leibniz Institute for the Analysis of Biodiversity Change (LIB), Museum of Nature-Zoology, Hamburg, 20146, Germany; 18 X-STAR, Japan Agency for Marine-Earth Science and Technology, Yokosuka, Kanagawa, Japan; 19 Comparative Genomics Laboratory, National Institute of Genetics, Mishima, Shizuoka, 411-8540, Japan; 20 School of Marine Science and Technology, Tokai University, Shizuoka, Shizuoka, Japan; 21 Ushimado Marine Institute, Graduate School of Natural Science and Technology, Okayama University, Setouchi, Japan., Okayama, Japan; 22 Highly Migratory Resources Division, Fisheries Resources Institute, Japan Fisheries Research and Education Agency, Shizuoka, Shizuoka, Japan; 23 Aquamarine Fukushima, Iwaki, Fukushima, Japan; 24 Marine Science Museum, Tokai University, Shizuoka, Shizuoka, Japan; 25 Osaka Aquarium Kaiyukan, Osaka, Osaka, Japan; 26 Laboratory of Physiology, Atmosphere and Ocean Research Institute, University of Tokyo,, Kashiwa, Chiba, Japan; 27 Department of Life Sciences, Graduate School of Arts and Sciences, The University of Tokyo, Tokyo, Tokyo, Japan

**Keywords:** Shark, ray, chimaera, biodiversity genomics, whole genome sequencing, karyotype

## Abstract

The taxon Elasmobranchii (sharks and rays) contains one of the long-established evolutionary lineages of vertebrates with a tantalizing collection of species occupying critical aquatic habitats. To overcome the current limitation in molecular resources, we launched the Squalomix Consortium in 2020 to promote a genome-wide array of molecular approaches, specifically targeting shark and ray species. Among the various bottlenecks in working with elasmobranchs are their elusiveness and low fecundity as well as the large and highly repetitive genomes. Their peculiar body fluid composition has also hindered the establishment of methods to perform routine cell culturing required for their karyotyping. In the Squalomix consortium, these obstacles are expected to be solved through a combination of in-house cytological techniques including karyotyping of cultured cells, chromatin preparation for Hi-C data acquisition, and high fidelity long-read sequencing. The resources and products obtained in this consortium, including genome and transcriptome sequences, a genome browser powered by JBrowse2 to visualize sequence alignments, and comprehensive matrices of gene expression profiles for selected species are accessible through https://github.com/Squalomix/info.

## Introduction

**Figure 1.**
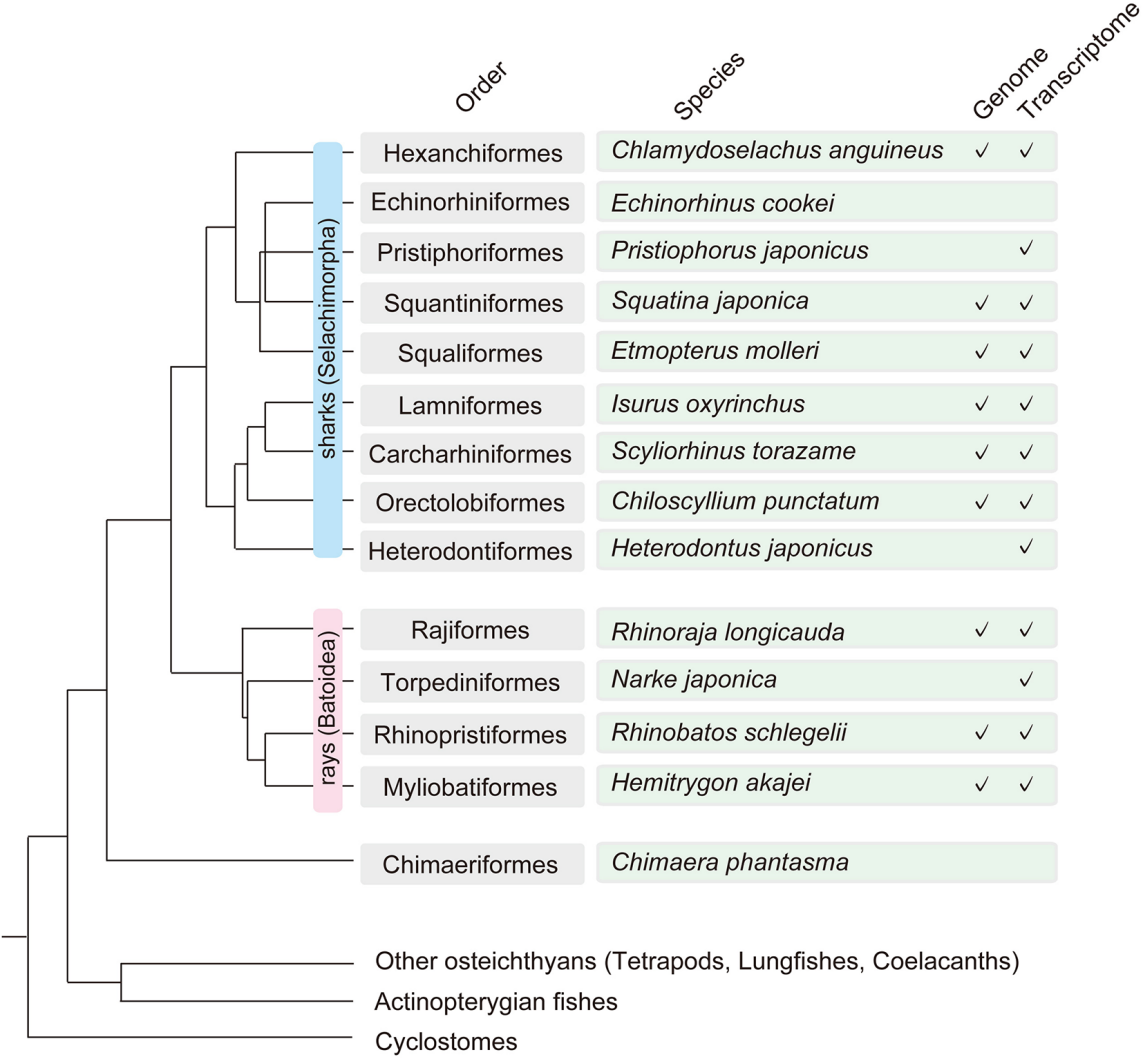
Chondrichthyan phylogeny and taxon sampling in the Squalomix Consortium. This figure includes some chondrichthyan species selected to represent the individual taxonomic orders that reflect the local fauna of Japan and are/will be analyzed by the consortium by genome or transcriptome sequencing (as of April 10, 2022). The full list of species and current status can be found in https://github.com/Squalomix/info.

Although usually recognized as a kind of ‘fish’ like actinopterygian fishes, cartilaginous fishes (chondrichthyans) form a distinct class of vertebrates with more than 1,200 species, known mostly as sharks and rays ( Figure 1; Nelson *et al.*, 2016). This taxonomic class has the longest evolutionary history among vertebrates of about 400 million years, in terms of the divergence of extant members ( Naylor *et al.*, 2012). Whereas its diversity might not be widely recognized, species in this taxon are characterized by several unique traits including electromagnetic sensing (all cartilaginous fishes), electricity generation (electric rays), diverse morphology sometimes with a flattened body (angelsharks and most rays) and/or a toothed rostrum (sawsharks and sawfishes). The highlight of their biological enigmas is in their reproductive modes with high plasticity between oviparity and viviparity, and occasionally parthenogenesis and intersexuality ( Penfold and Wyffels, 2019). Mainly because of overfishing, many cartilaginous fish populations are declining ( Pacoureau *et al.*, 2021), and evidence-based resource management would greatly benefit from the establishment of genomic platforms.

Despite these outstanding evolutionary and biological importance, modern genomic approaches have only recently been applied to cartilaginous fishes (reviewed in Kuraku, 2021). The only exception is the effort commenced before 2010 on the elephant fish *Callorhinchus milii* ( Venkatesh *et al.*, 2014), a member of the Holocephali (chimaeras and ratfishes), the more species-poor chondrichthyan lineage, with a relatively small genome size of about 1.9 giga basepairs (Gbp). In contrast, most elasmobranchs have genomes of more than 3 Gbp plagued with abundant repetitive elements.

## Squalomix: consortium scope and organization

**Figure 2.**
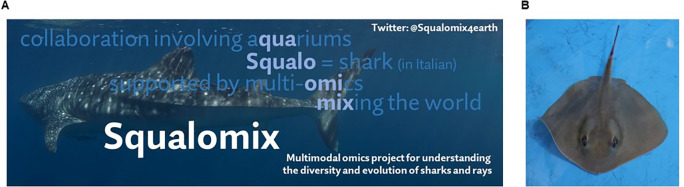
Squalomix Consortium. A, Consortium logo. B, One of the main study species, the red stingray *Hemitrygon akajei.* Photo credit: Itsuki Kiyatake.

The Squalomix Consortium ( Figure 2A) was launched in 2020 aiming to provide the genome sequence and other genome-wide data for chondrichthyan species including transcriptomes and epigenomes. Sample processing and data production is conducted by the Molecular Life History Laboratory at the National Institute of Genetics, Mishima, Japan, and the Laboratory for Phyloinformatics in RIKEN Kobe, Japan, which harbors a DNA Analysis Facility. The consortium is funded by academic agencies as of May 2022 and is seeking additional funding sources, especially from industrial groups oriented toward the conservation of biodiversity and marine environments. In November 2020, the Squalomix Consortium became affiliated with Earth BioGenome Project (EBP), the global initiative to promote biodiversity genomics ( Lewin *et al.*, 2022). The collaborative network at the Squalomix Consortium includes an extensive range of expertise and worldwide distribution.

## Versatile sample collection featuring the local fauna

In Squalomix, sample collection is performed cautiously to minimize the sacrifice of wildlife—especially those with an endangered status. The collection focuses mainly on the rich marine fauna in Japan’s neighboring temperate waters, with occasional sources from death stranding for elusive species. The project collaborates closely with local aquariums oriented toward academic science. Their contributions play indispensable roles in relaying offshore sampling and enable sustainable sampling of embryos and blood from live individuals, although the latter approach is limited to species that can be bred in captivity and are amenable to husbandry.

**Figure 3.**
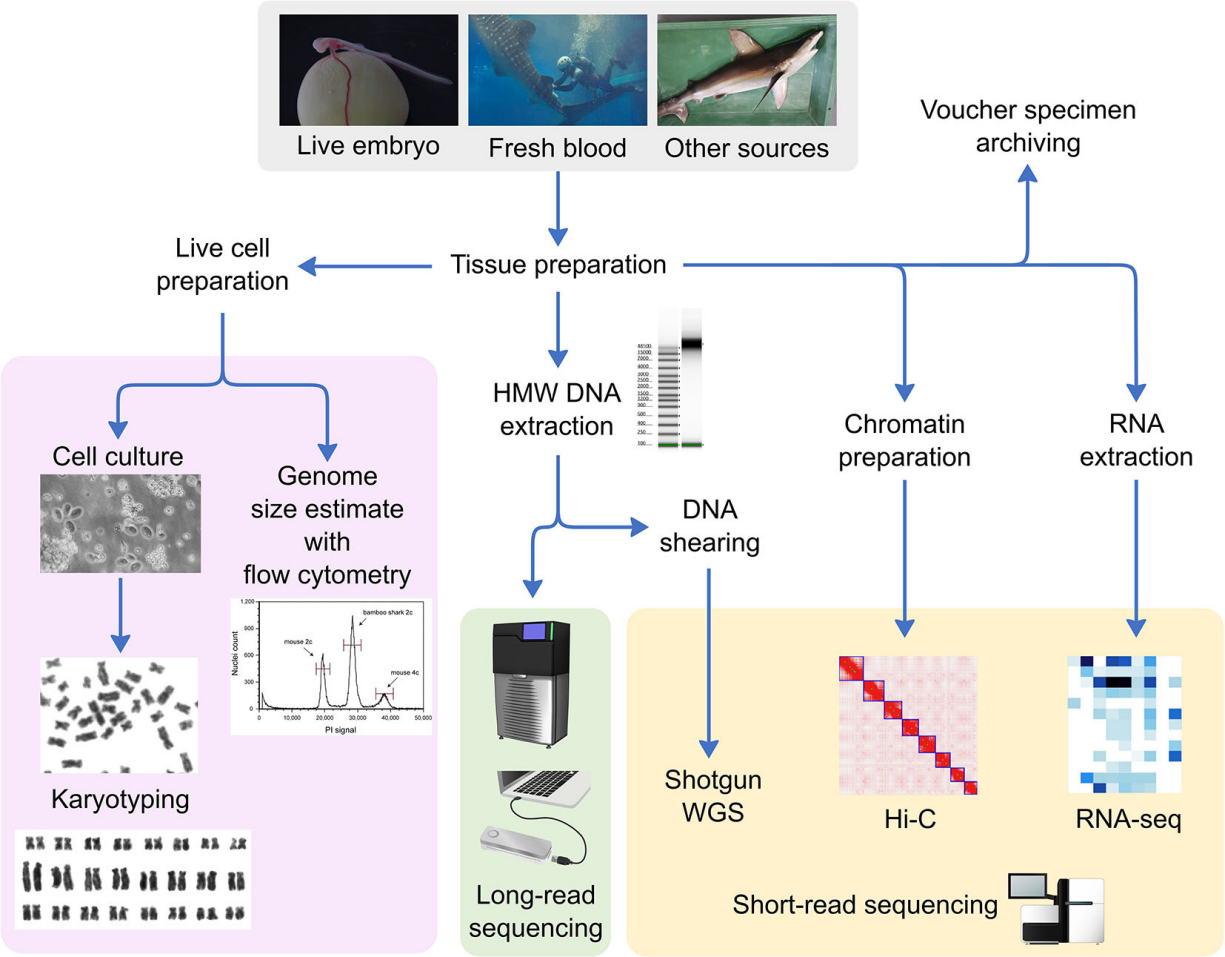
Typical work flow in the Squalomix Consortium. Whole genome sequencing (WGS) is mainly performed with the Sequel II/IIe platform (Pacific Biosciences, Inc.) to obtain high-fidelity (HiFi) long reads, which is supplemented by short-read sequencing. Extraction of high molecular weight (HMW) genomic DNA is mainly performed using the NucleoBond columns (Macherey-Nagel, Inc.) and the extracted DNA is controlled with Agilent TapeStation systems (Agilent Technologies, Inc.) as well as conventional pulse-field gel electrophoresis. Flow cytometry for genome size estimation employs the Ploidy Analyser platform (Sysmex Inc.). Hi-C sample preparation employs the iconHi-C protocol ( Kadota *et al.*, 2020) that was optimized in-house based on several existing protocols.

Another strength of the Squalomix Consortium is its expertise in laboratory solutions that are not confined to DNA sequencing, but additionally explore post-genome approaches to decipher the molecular basis of chondrichthyan phenotypic evolution. Access to fresh tissues from local aquaria facilitates embryological analysis, genome size quantification with flow cytometry, and karyotyping from cell cultures ( Figure 3). Remarkably, cell culture in cartilaginous fishes, which was long thought difficult because of their high body fluid osmolarity, was enabled by modifying the culture medium with balancing osmolytes ( Uno *et al.*, 2020). Our cytological expertise also allowed various epigenomic analyses that benefit from whole genome sequencing, on transcription factor binding with ChIP-seq ( Hara *et al.*, 2018) and chromatin openness with ATAC-seq, in addition to long-range DNA interactions with Hi-C ( Kadota *et al.*, 2020; Onimaru *et al.*, 2021). These techniques contributed to biological analyses based on the draft genome sequences of three shark species ( Hara *et al.*, 2018), which launched the Squalomix Consortium.

## Sequencing strategy and recent progress

**Figure 4.**
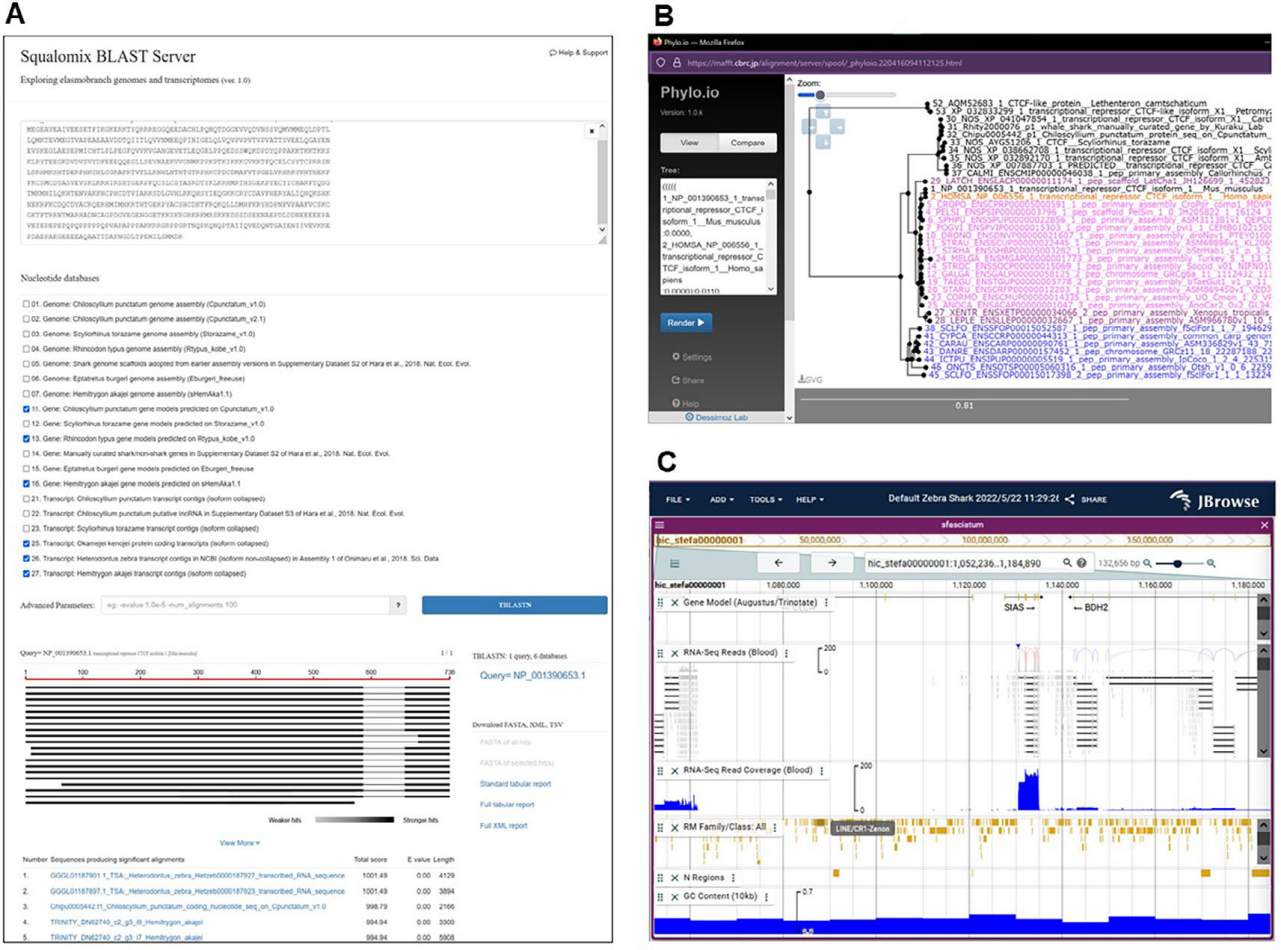
Overview of the Squalomix data sharing platform. A, Sequence similarity search (BLAST) in elasmobranch genome and transcriptome sequences. B, Molecular phylogeny inference facilitated by the existing combination of aLeaves (that hosts products of Squalomix) and MAFFT webservers ( Kuraku *et al.*, 2013). C, Interactive genome browser employing JBrowse2 version 1.6.9 ( Buels *et al.*, 2016) for the zebra shark *Stegostoma tigrinum* (or *S. fasciatum*) based on its first genome assembly sSteFas1.1 (NCBI Genome ID, GCA_022316705.1). The websites providing these functions are found through the main consortium gateway ( https://github.com/Squalomix/info).

The sequencing strategy in the Squalomix Consortium is designed to accommodate genomic characteristics of cartilaginous fishes, mostly with large, repetitive genomes. In the standard protocol formulated in January 2021 ( Figure 3), we start by estimating genome size using flow cytometry and karyotyping as well as by ‘survey’ sequencing of transcriptomes, which serves for species identity verification with an assembled mitochondrial DNA sequence. These initial steps ensure sample authenticity and quality. We then proceed to genome sequencing, which employs both short-read and long-read high-fidelity (‘HiFi’) sequencing platforms, together with Hi-C data production for chromosome-scale scaffolding based on three-dimensional DNA interactions. The long-read data are obtained using the Sequel II or IIe platforms (Pacific Biosciences, Inc.) with a minimum sequencing depth of 20x. The assembly outputs are evaluated with reference to their coverage of protein-coding gene space, as well as transcriptome data, genome size, and karyotypic organization obtained separately. These validations allow us to scrutinize the inclusion of those genomic regions that are difficult to sequence and assemble, such as the Hox C genes that were previously thought to be missing in elasmobranchs but were retrieved by elaborate annotation ( Hara *et al.*, 2018; reviewed in Kuraku, 2021). Complete genome assemblies are critical to validate gene loss and variations in gene repertoires via synteny/phylogeny comparisons, previously suggested for visual opsins and conventional olfactory receptors ( Hara *et al.*, 2018). The standard procedure outlined above ( Figure 3) has been applied to several study species, including the red stingray *Hemitrygon akajei* ( Figure 2B) for which a draft genome assembly has been made available for BLAST searches at the Squalomix sequence archive ( Figure 4A; https://transcriptome.riken.jp/squalomix/).

## Cooperation toward the global goals

The Squalomix Consortium aims not only to sequence and analyze the genomes but also to tightly interact with other research groups whose target species list contains cartilaginous fishes including other EBP-affiliated projects (see below). To maximize mutual benefit among those projects, some animal samples from our collection could be provided for genome sequencing at other sites. The Squalomix Consortium offers laboratory experiments for genome size quantification or karyotype analysis for species listed by other consortia, provided that fresh cells are available. The sample transfer will be processed in accordance with the Nagoya Protocol and other relevant regulations. Inclusive cooperation respecting complementary expertise is expected to overcome the long-standing difficulty in studying elasmobranchs sustainably and contribute to disentangling the marine ecosystems for effective conservation.

## Data sharing platforms

Once produced, genome assemblies pass rigid quality controls and are deposited in the NCBI Genome under the NCBI BioProject ID PRJNA707598 and made available as database for BLAST searches at our Squalomix sequence archive ( https://transcriptome.riken.jp/squalomix/). This archive also has a link to the up-to-date listing of the species for which genome sequences are available, filed by the GenomeSync database ( http://genomesync.org/). The archive website also hosts a gateway to genome browsers powered by JBrowse2 that allow users to visualize specific genomic regions and load additional tracks including base composition, gene models, repetitive elements, and aligned RNA-seq reads ( Figure 4C). We also provide comprehensive matrices of expression profiles for predicted genes of the brownbanded bamboo shark *Chiloscyllium punctatum* and the cloudy catshark *Scyliorhinus torazame* that were already quantified and normalized based on RNA-seq data of various tissues for our past publication ( Hara *et al.*, 2018).

## Other pioneering efforts tackling elasmobranch genomes

Some elasmobranch genomes have already been sequenced by other pioneering working groups ( https://www.ncbi.nlm.nih.gov/data-hub/genome/?taxon=7777&reference_only=true). This includes the Vertebrate Genomes Project (VGP), whose data production format employs a suite of modern promising solutions including optical mapping and Hi-C scaffolding as well as long-read and short-read sequencing, to cover all vertebrate species ( Rhie *et al.*, 2021). The initial VGP progress report released the genome sequences of the thorny skate *Amblyraja radiata* (NCBI Genome ID, GCA_010909765.2). The Darwin Tree of Life (DToL) Project partly links with VGP and aims to sequence all eukaryotic species in Britain and Ireland. DToL’s first chondrichthyan genome is that of the small-spotted catshark *Scyliorhinus canicula,* the egg-laying species most widely studied in developmental biology and endocrinology (NCBI Genome ID, GCA_902713615.1). The recently launched European Reference Genome Atlas (ERGA) also plans to produce reference chromosome anchored genomes of multiple species from this geography including cartilaginous fish aiming to empower conservation efforts ( Formenti *et al.*, 2022). Researchers in China launched the Fish10K project that partially targets cartilaginous fishes ( Fan, *et al.*, 2020). In addition, the DNA Zoo project puts special emphasis on Hi-C scaffolding ( Rao *et al.*, 2014), often using available genome assemblies already released by other groups as input and performing chromosome-scale genome scaffolding using Hi-C data even in the presence of intra-specific genomic variations. So far, the DNA Zoo effort produced the chromosome-scale genome assemblies of the brownbanded bamboo shark *C. punctatum* and the whale shark *Rhincodon typus*, each of which was produced using samples from multiple individuals ( Hoencamp *et al.*, 2021). All the above efforts are expected to be coordinated under the overarching EBP initiative, in order to play complementary roles towards the global aim of generating high-quality genomic resources.

## Data availability

Products from this consortium are deposited in NCBI under the BioProject ID PRJNA707598 and are available at our Squalomix sequence archive ( https://transcriptome.riken.jp/squalomix/).
