# Supplementary material for: Squalomix: shark and ray genome analysis consortium and its data sharing platform
Source: F1000Res. 2022 Sep 21;11:1077. [Version 1] doi: 10.12688/f1000research.123591.1 (PMC9561540; doi:10.12688/f1000research.123591.1)
Supplement: Peer review history

# Peer review: Reviewer response for version 1

**DOI:** 10.5256/f1000research.135712.r151219

**Published:** 2022-10-13

**Authors:** Dan Larhammar, David Lagman

**Affiliations:** 1 Department of Neuroscience, Science for Life Laboratory, Uppsala University, Uppsala, Sweden; 2 Uppsala University, Uppsala, Sweden

I greet this initiative with great enthusiasm. The description is well written, clear and easy to follow. I have just a few comments that I hope the authors will consider.

 In the introduction, the authors describe Chondrichthyes as the oldest vertebrate class ("longest evolutionary history"). However, this is due to the imprecise use of the term "class" in vertebrate taxonomy where both Chondrichthyes and Mammalia are designated as classes. Thus, classes are not of equal temporal rank. Furthermore, the authors' statement is not quite true, because even Agnatha has the taxonomic rank as a vertebrate class and would thereby be even earlier than Chondrichthyes. I would recommend the authors to describe Chondrichthyes instead as one of the two lineages resulting from the first bifurcation or divergence in (the infraphylum of) Gnathostomata (jawed vertebrates). This, by the way, means that Osteichthyes is as old as Chondrichthyes!

 Please correct the grammar of the expression "Despite these outstanding evolutionary and biological importance…". It probably needs to be rephrased, perhaps like this:

 "Despite the outstanding evolutionary and biological importance of chondrichthyans…" (or elasmobranchs if you prefer to focus on these).

 Shouldn't "giga basepairs" be one word as one would surely write megabasepairs and kilobasepairs.

 Figure 1: The expression "Other osteichthyans" is imprecies. I assume the authors want to avoid the term "Sarcopterygians" for this group pterygi means fins, and tetrapods of course don't have them. Maybe it's better to just delete "Other osteichthyans" and let this branch be called "Tetrapods, lungfishes and coelacanths) (without capital initial letters).

Are sufficient details of methods and materials provided to allow replication by others?

Yes

Is the rationale for creating the dataset(s) clearly described?

Yes

Are the datasets clearly presented in a useable and accessible format?

Yes

Are the protocols appropriate and is the work technically sound?

Yes

Reviewer Expertise:

Gene family evolution, pharmacology of G protein-coupled receptors, mechanism of long-term memory

We confirm that we have read this submission and believe that we have an appropriate level of expertise to confirm that it is of an acceptable scientific standard.

# Peer review: Reviewer response for version 1

**DOI:** 10.5256/f1000research.135712.r151220

**Published:** 2022-09-26

**Authors:** Mélanie Debiais-Thibaud

**Affiliations:** 1 Institut des Sciences de l’Evolution de Montpellier, ISEM, Université Montpellier, Montpellier, France

In this Data note, the authors describe and wrap-up all available material generated through their consortium named Squalomix, in which a set of biological material and sequence data obtained in elasmobranch organisms are made available to the research community. The rationale, protocol, material and data availability are clearly described in this Note.

Are sufficient details of methods and materials provided to allow replication by others?

Yes

Is the rationale for creating the dataset(s) clearly described?

Yes

Are the datasets clearly presented in a useable and accessible format?

Yes

Are the protocols appropriate and is the work technically sound?

Yes

Reviewer Expertise:

Developmental genetics, EvoDevo

I confirm that I have read this submission and believe that I have an appropriate level of expertise to confirm that it is of an acceptable scientific standard.
